# Characterization and Comparison of Two Complete Plastomes of Rosaceae Species (*Potentilla dickinsii* var. *glabrata* and *Spiraea* *insularis*) Endemic to Ulleung Island, Korea

**DOI:** 10.3390/ijms21144933

**Published:** 2020-07-13

**Authors:** JiYoung Yang, Gi-Ho Kang, Jae-Hong Pak, Seung-Chul Kim

**Affiliations:** 1 Research Institute for Dok-do and Ulleung-do Island, Department of Biology, School of Life Sciences, Kyungpook National University, 80 Daehak-ro, Buk-gu, Daegu, Gyeongsangbuk-do 41566, Korea; whity@daum.net; 2 Baekdudaegan National Arboretum, 1501 Chunyang-ro, Chungyang-myeon, Bonghwa-gun, Gyeongsangbuk-do 36209, Korea; supia@kiam.or.kr; 3 Department of Biological Sciences, Sungkyunkwan University, 2066 Seobu-ro, Suwon, Gyeonggi-do 16419, Korea

**Keywords:** *Potentilla dickinsii* var. *glabrata*, *Spiraea insularis*, Rosaceae, Ulleung Island, plastome, anagenetic speciation

## Abstract

*Potentilla dickinsii* var. *glabrata* and *Spiraea insularis* in the family Rosaceae are species endemic to Ulleung Island, Korea, the latter of which is listed as endangered. In this study, we characterized the complete plastomes of these two species and compared these with previously reported plastomes of other Ulleung Island endemic species of Rosaceae (*Cotoneaster wilsonii*, *Prunus takesimensis*, *Rubus takesimensis*, and *Sorbus ulleungensis*). The highly conserved complete plastomes of *P. dickinsii* var. *glabrata* and *S. insularis* are 158,637 and 155,524 base pairs with GC contents of 37% and 36.9%, respectively. Comparative phylogenomic analysis identified three highly variable intergenic regions (*trnT*-UGU/*trnL*-UAA, *rpl32/trnL*-UAG, and *ndhF/rpl32*) and one variable genic region (*ycf1*). Only 6 of the 75 protein-coding genes have been subject to strong positive selection. Phylogenetic analysis of 23 representative plastomes within the Rosaceae supported the monophyly of *Potentilla* and the sister relationship between *Potentilla* and *Fragaria* and indicated that *S. insularis* is sister to a clade containing *Cotoneaster*, *Malus*, *Pyrus*, and *Sorbus*. The plastome resources generated in this study will contribute to elucidating the plastome evolution of insular endemic Rosaceae on Ulleung Island and also in assessing the genetic consequences of anagenetic speciation for various endemic lineages on the island.

## 1. Introduction

The family Rosaceae (Rosid I, Fabidae) comprises approximately 3000 species in 91 genera within three subfamilies (Rosoideae, Amygdaloideae, and Dryadoideae) and includes numerous economically important commercial fruit species, such as *Malus* (apples), *Prunus* (almonds, plums, peaches, cherries, etc.), *Pyrus* (pears), *Rubus* (raspberries, blackberries), and *Fragaria* (strawberries) [1,2,3]. Numerous ornamental woody species, such as crabapples (*Malus*), hawthorns (*Crataegus*), roses (*Rosa*), and rowans (*Sorbus*), are also valued members of this family. In addition to being a source of important commercial fruit and ornamental/landscaping trees, woody species of Rosaceae provide important habitats and food resources for innumerable forest animals, including birds and mammals [2]. The family Rosaceae is particularly diverse in temperate regions of the Northern Hemisphere and in this regard, it has been suggested that several closely related factors, including hybridization, pseudogamous gametophytic apomixes, polyploidy, and self-compatibility, have been driving forces that have contributed to promoting increases in the number of species in certain groups [1,4,5,6]. Given its considerable economic and taxonomic significance, the family Rosaceae has been subject to numerous genomic and phylogenetic studies [1,6,7,8,9,10]. Among the numerous important contributions to the phylogenetic characterization of Rosaceae, the whole plastid phylogenomic approach, based on 142 accessions, representing 87 genera, reported by Zhang et al., is of particular interest because they attempted to reconstruct deep relationships and reveal temporal diversification of the family [10]. In addition, on the basis of 125 new transcriptomic and genomic datasets, Xiang et al. fully resolved the phylogeny of Rosaceae, revealing numerous whole-genome duplications and providing a foundation for understanding the evolution of fruit-bearing plants in the Rosaceae [6].

Ulleung Island is located approximately 137 km off the east coast of the Korean Peninsula and approximately 300 km west of the Japanese archipelago. The island is estimated to be approximately 1.8 million years old and has a total area of 73 km^2^ [11]. Ulleung Island is home to some 500 native vascular plants species, of which approximately 40 (nearly 8%) are endemic, and is renowned for the exceptionally high number of plant species that evolved via anagenetic speciation [12,13]. Thus, the endemic plants of Ulleung Island represent an ideal model system for investigating the mechanism of anagenetic speciation and evaluating its genetic consequences [14,15,16,17,18,19,20]. Of the nearly 40 endemic species inhabiting Ulleung Island, Rosaceae includes the largest number of diverse endemics. In addition to having autapomorphies for each species, these Ulleung endemics show a general trend toward increasing leaf and flower size and loss of spines, which may be related to an absence of herbivores on the island [12,15].

**Figure 1:**
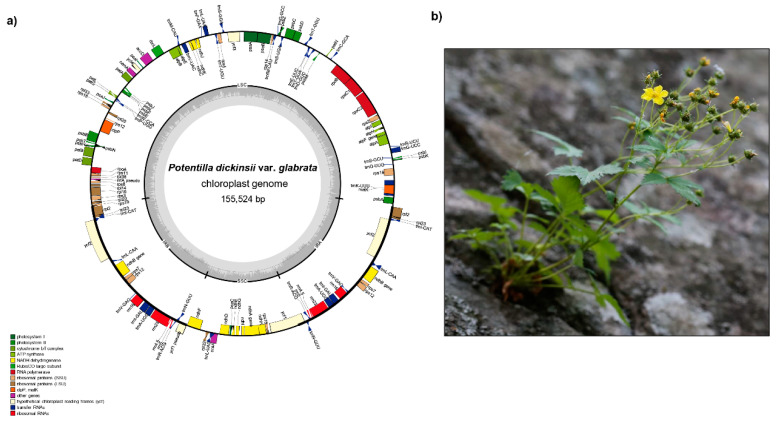
The complete plastome map (**a**) and plant of *Potentilla dickinsii* var. *glabrata* (**b**). (Photograph credit: Jin-Oh Hyun, Northeastern Asia Biodiversity Institute, Korea).

**Figure 2:**
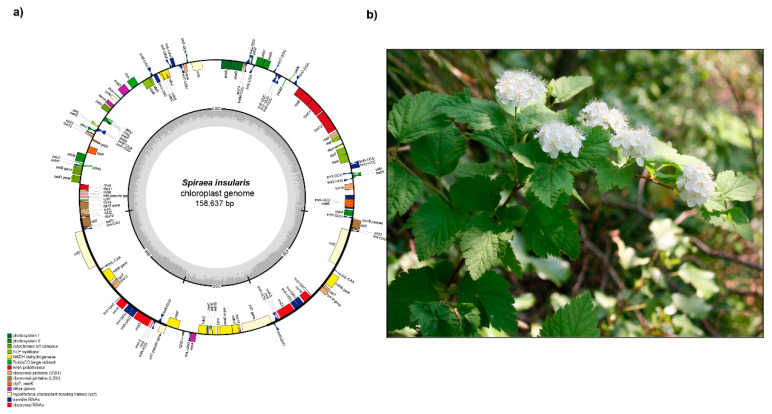
The complete plastome map (**a**) and plant of *Spiraea insularis* (**b**). (Photograph credit: Seung-Chul Kim, Sungkyunkwan University, Korea).

*Spiraea insularis* (=*Physocarpus insularis*), belongs to the subfamily Amygdaloideae and is found rarely in the southeastern part of Ulleung Island [1,21,22,23,24] (Figure 1). Given its extreme rarity and highly restricted distribution, *S. insularis* is categorized and protected as a critically endangered species [CRB2ab(ii)] by the Korean government [25]. The classification and taxonomic position of this species has continued to prove problematic. For example, Kim et al. [26] treated it as a synonym of *Spiraea chamaedryfolia* var. *ulmifolia*, but various floristic studies have all identified *P. insularis* as a distinct species [27,28,29]. Given the established intercontinental disjunct distribution of *Physocarpus* [30] and that *P. insularis* is the only *Physocarpus* species native to Korea, its taxonomic status had remained uncertain until Oh et al. [31] confirmed its phylogenetic position within the genus *Spiraea*. Furthermore, on the basis of a few autapomorphic morphological characteristics, *P. insularis* was subsequently transferred and recognized as the new combination *S. insularis* (Nakai) H. Shin, Y.D. Kim and S.H. Oh [32]. The other Rosaceous endemic *Potentilla dickinsii* var. *glabrata*, a perennial herb, belongs to the subfamily Rosoideae sensu [1] (Figure 2). It was originally described as a new taxon by Nakai and is closely related to *P. dickinsii* var. *dickinsii*, which is distributed on the mainland of Korea and Japan [21]. However, Naruhashi [33] treated this taxon as a synonym of *P. dickinsii* var. *dickinsii* based on the wide infraspecific variation of *P. dickinsii*. Nevertheless, it has been suggested that *P. dickinsii* var. *glabrata* should be recognized as a distinct taxon based on its well-developed thick rhizomes and trifoliate basal leaves that differ from the typically pinnately compound leaves found in var. *dickinsii* [34]. It is widely acknowledged that species within the genus *Potentilla* are taxonomically a very challenging group, owing to hybridization, allopolyploidy, and apomixes [35,36,37].

The chloroplast genome can provide ample and vital information regarding genetic variation at the different taxonomic levels, particularly with respect maternal genetic characteristics, given its typical uniparental mode of inheritance in angiosperms [38]. Plastomes have accordingly been efficiently utilized to infer the phylogenetic relationships and evolutionary histories of numerous plant groups [10,39,40,41]. Since the first report of the complete plastome sequences of strawberry (*Fragaria vesca*) and apple (*Malus* × *domestica*), the plastome sequences of numerous species in the Rosaceae have been characterized over the past decade, which has been markedly facilitated by the advent of next-generation sequencing (NGS) technology [42,43,44,45,46,47,48,49]. Recently, complete plastome sequences have also been reported for several plant species among the diverse groups on Ulleung Island, including *Campanula takesimana* (Campanulaceae) [50], *Chrysanthemum lucidum* (Asteraceae) [51], *Fagus multinervis* (Fagaceae) [52], *Lilium hansonii* (Liliaceae) [53], *Tilia insularis* (Malvaceae) [54], *Epilobium ulleungensis* (Onagraceae) [55], *Phytolacca insularis* (Phytolaccaceae) [56], and *Acer takesimense* (Sapindaceae) [57]. Moreover, complete plastome sequences have been obtained for four species of Rosaceae endemic to Ulleung, namely, *Rubus takesimensis*, *Prunus takesimensis*, *C. wilsonii*, and *Sorbus ulleungensis*, which have provided important information regarding plastome organization and evolution [47,48,58,59]. 

In this study, we characterized the complete chloroplast genome sequences of *Potentilla dickinsii* var. *glabrata* (subfamily Rosoideae) and *Spiraea insularis* (subfamily Amygdaloideae), which are the only Rosaceae species endemic to Ulleung for which plastomes are yet to be sequenced, and compared these with the plastomes of the aforementioned four Ulleung Rosaceae species. The comparative analysis of these six plastomes will shed light on the plastome structure and evolution of endemic insular species in the family Rosaceae, which have evolved through the speciation mechanism of anagenesis. We anticipate that further analyses of these plastome sequences will enable us to identify hotspot regions that contribute to determining population genetic diversity and structure, thereby allowing us to assess genetic differences between pairs of continental progenitor and insular derivative species.

## 2. Results and Discussion

### 2.1. Genome Size and Features

The plastome of *P. dickinsii* var. *glabrata* has 155,524 bp and comprises a large single-copy (LSC) region of 85,213 bp, a small single-copy (SSC) region of 18,657 bp, and two inverted repeat (IR) regions of 25,827 bp. The complete plastome sequence of *S. insularis* is slightly larger at 158,637 bp and comprises an LSC region of 86,997 bp, an SSC region of 18,910 bp, and two IR regions of 26,365 bp (Figure 1 and Figure 2, and Table 1). The plastomes of *P. dickinsii* var. *glabrata* and *S. insularis* contain 131 and 132 genes, respectively, with the difference in gene number being attributable to the presence of an *rps19* pseudogene in *S. insularis*. Both plastomes contain 84 protein-coding, eight ribosomal RNA, and 37 transfer RNA genes. The overall guanine–cytosine (GC) content of the *P. dickinsii* var. *glabrata* and *S. insularis* plastomes are 37.0% and 36.91%, respectively (Table 1). Of the six Rosaceae endemic to Ulleung Island, *C. wilsonii* is characterized by the longest plastome (159,997 bp), whereas that of *P. dickinsii* var. *glabrata* is the shortest. The plastomes of *R. takesimensis* and *Sorbus ulleungensis* were found to have the highest and lowest GC content of 37.1% and 36.5%, respectively. The plastome sequences of both *P. dickinsii* var. *glabrata* and *S. insularis* were found to contain a total of 17 duplicated genes in the IR regions (seven tRNA, four rRNA, and six protein-coding genes). Fifteen genes (*ndhA*, *ndhB*, *petB*, *petD*, *rpl2*, *rpl16*, *rpoC1*, *rps12*, *rps16*, *trnA*-UGC, *trnG*-UCC, *trnI-*GAU, *trnK-*UUU, *trnL-*UAA, and *trnV-*UAC) contain a single intron, whereas *clpP* and *ycf3* each contain two introns.

In both *P. dickinsii* var. *glabrata* and *S. insularis*, the plastome contains a partial *ycf1* gene of 1227 and 1301 bp, respectively, located in the IRb/SSC junction region, whereas a complete *ycf1* gene of 5808 and 5613 bp, respectively, is located in the IR region at the SSC/IRa junction. The *infA* gene located in the LSC region of the *P. dickinsii* var. *glabrata* and *S. insularis* has become a pseudogene. Interestingly, the highly conserved group II intron of *atpF* has been lost in *S. insularis*, as has previously been observed in the *Rubus* species *R. boninensis*, *R. crataegifolius*, *R. takesimensis*, and *R. trifidus* [47,48,60]. When compared with representative plastomes of species in the Rosaceae, those of *Potentilla*, *Fragaria*, *Rosa*, and *Rubus* in the subfamily Rosoideae lineage all show *atpF* intron loss, whereas in contrast, the plastomes of *Cotoneaster*, *Malus*, *Prunus*, *Pyrus*, and *Sorbus* in the subfamily Amygdaloideae retain intron-containing *atpF* genes (Table 1). On the basis of the current phylogenetic framework, it appears that loss of the *atpF* intron has occurred only once in the subfamily Rosoideae; however, it remains to be determined whether this loss has also occurred in other lineages of Rosoideae, as well as in the broader phylogenetic framework, including within the Rosacea and Rosid families.

**Figure 3:**
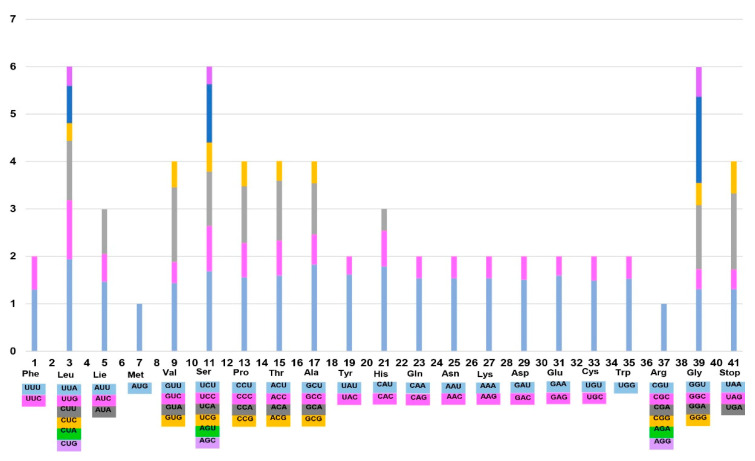
Codon distribution and relative synonymous codon usage in the plastomes of two Rosaceae species endemic to Ulleung Island.

The frequency of codon usage in the *P. dickinsii* var. *glabrata* and *S. insularis* plastomes was calculated for the chloroplast genome based on the sequences of protein-coding and tRNA genes (Figure 3), which revealed that the average codon usage in these two species was nearly identical, i.e., 26,008 for *P. dickinsii* var. *glabrata* and 26,015 for *S. insularis*. Moreover, we found the distribution of codon types to be consistent. The relative synonymous codon usage (RSCU) value was also similar to that in *R. takesimensis*. Consistent with the patterns detected in *Rubus* [60] and other angiosperms [61] and algal lineages [62], we found that codon usage in the *P. dickinsii* var. *glabrata* and *S. insularis* plastomes is biased toward a high RSCU value of U and A at the third codon position.

The predicted number of RNA editing sites in the plastomes of *P. dickinsii* var. *glabrata* and *S. insularis* is 42 and 51, respectively, with the same cut-off value, and 19 and 15 of 35 protein-coding genes are predicted to undergo RNA editing, respectively (Table S1). These genes include photosynthesis-related genes (*atpF*, *atpI*, *ndA*, *ndhB*, *ndhD*, *ndhF*, *ndhG*, *petB*, *petG*, *psbE*, and *psbF*), self-replication genes (*rpl23*, *rpoA*, *rpoB*, *rpoC2*, *rps2*, *rps14*, and *rps16*), and others (*accD*, *clpP*, and *matK*). We detected no RNA editing sites in the *accD*, *atpF*, *atpI*, *psbE*, *psbF*, and *rps16* genes of the *P. dickinsii* var. *glabrata* plastome, whereas no RNA editing sites were found at *petG* and *rpl23* in the *S. insularis* plastome. Compared with other species, the *ndhF* gene of *S. insularis* showed an exceptionally high frequency (i.e., three-fold higher) of RNA editing sites. The *ndhB* gene is also characterized by the highest number of potential editing sites (11 sites), followed by the *ndhD* gene (6 sites), which is consistent with the findings of previous studies [63,64,65].

### 2.2. Comparative Analysis of Genome Structure

**Figure 4:**
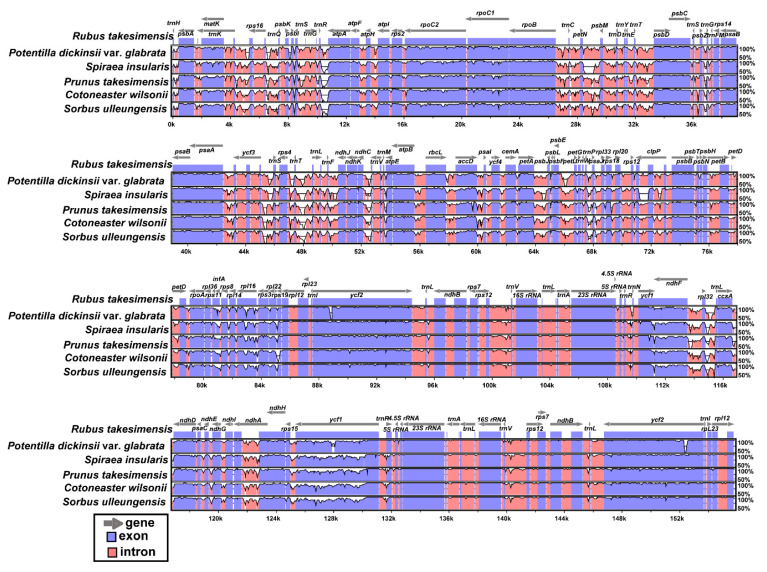
Visualization of alignment of the six plastome sequences of Rosaceae species endemic to Ulleung Island.

The plastomes of the six Rosaceae species endemic to Ulleung Island (i.e., *C. wilsonii*, *S. insularis*, *P. dickinsii* var. *glabrata*, *Prunus takesimensis*, *R. takesimensis*, and *Sorbus ulleungensis*) were plotted with mVISTA, using the annotated *R. takesimensis* plastome as a reference (Figure 4). The results indicated that the LSC region is the most divergent, whereas the two IR regions are highly conserved. In addition, the non-coding regions were found to be more divergent and variable than the coding regions. As expected, these findings are consistent with the patterns observed in common angiosperms [44,47,49,60,61]. These six plastomes are highly conserved despite differences in estimated divergence times in the Late Cretaceous period, with the crown ages of Rosoideae and Amygdaloideae being estimated to be 75.78 million and 90.18 million years, respectively [10].

**Figure 5:**
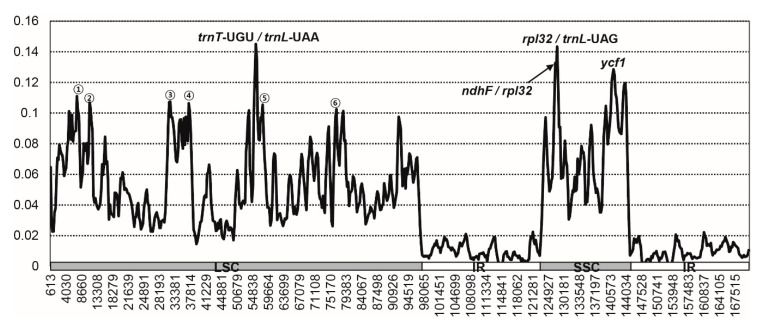
Sliding window analysis of the six whole-chloroplast genomes of Rosaceae species endemic to Ulleung Island: 1—*rps16*/*trnQ*-UUG; 2—*trnR*-UCU/*atpA*; 3—*rpoB*/*trnC*-GCA/*petN*; 4—*trnT*-GGU/*psbD*; 5—*ycf3*/*trn*-GGA/*rps4*; and 6—*trnP*-UGG/*psaJ*/*rpl33*.

Sliding window analysis performed using the DnaSP program revealed highly variable regions in the plastomes of the six endemic Rosaceae taxa (Figure 5). Comparison of the six plastomes revealed that the average value of nucleotide diversity (*Pi*) over the entire chloroplast genome was 0.042, with the most variable region (a *Pi* value of 0.14508) being the *trnT-*UGU/*trnL-*UAA intergenic region. We also detected high variability in two other intergenic regions (*rpl32/trnL-*UAG (*Pi* = 0.14342) and *ndhF/rpl32* (*Pi* = 0.13267)) and one genic region (*ycf1* (*Pi* = 0.1285)). In addition, we detected several variable regions with *Pi* values greater than 0.1, namely, *rps16/trnQ-*UUG, *trnR-*UCU*/atpA*, *rpoB/trnC-*GCA*/petN*, *trnT-*GGU*/psbD*, *trnP-*UGG*/psaJ/rpl33*, and *ycf3/trnS-*GGA*/rps4*. Among these highly variable regions, those with *Pi* values greater than 0.12 can be used to generate chlorotype diversity data to infer the origin and evolution of endemic species on Ulleung Island. Although the two newly sequenced species are only rarely found on Ulleung Island, *R takesimensis* is among the more commonly occurring species on the island, and we similarly detected highly variable regions, including *rpl32/trnL*, *rps4/trnT*, *trnT/trnL*, and *psbZ/trnG*, in this species [48]. Furthermore, we also found that the *ycf1* gene shows the highest sequence divergence and, thus, would appear to have potential value for the phylogenetic analysis of Rosaceae and angiosperms in general [46].

Positive selection analysis, performed using the EasyCodeML [66] program with the site-specific model based on CodeML algorithms [67], enabled us to identify positively selected genes among endemic Rosaceae on Ulleung Island (Table 2). Among the conserved genes, six genes with positively selected sites within the endemic Rosaceae plastomes on Ulleung Island were identified with effectively significant LRT *p* values (Table 2). These six genes include one subunit of acetyl-CoA carboxylase (*accD*), one Rubisco gene (*rbcL*), one ribosome small subunit gene (*rps3*) of self-replication, and three NADH-dehydrogenase subunit genes (*ndhB*, *nhdD*, and *ndhF*) of photosynthesis. Based on the M8 model, the *rbcL* gene had five positive sites, followed by *ndhF* (three sites), and *rps3* (two sites). The other three genes each had only one positive site. However, most of the genes, 69 of the 75 genes, had an average Ka/Ks ratio of below 1, indicating that these genes have been subjected to strong purifying selection in the Rosaceae chloroplast. In general, previous studies showed that Ka/Ks values are usually less than one [68], because synonymous nucleotide substitutions occur more frequently than nonsynonymous substitutions. Additionally, most genes of the chloroplast genome evolved under purifying selection due to functional limitation during chloroplast genome evolution [69,70,71,72]. Furthermore, both the positive selection of the *rbcL* gene and the NADH dehydrogenase subunit genes were previously reported in several studies, which is related to temperature, drought, carbon dioxide concentration, and photosynthetic rate [71,72,73,74]. Positive selection is considered to be indicative of an adaptation to environmental change, ecological niche, or coevolutionary processes [73,75], and we can, thus, speculate that the selection patterns detected for the Rosaceae endemic taxa on Ulleung Island may be associated with adaptation to an oceanic climate in the insular setting. However, any correlation between insular environment and positive selection pressures on genes will require further study.

### 2.3. Phylogenetic Analysis

**Figure 6:**
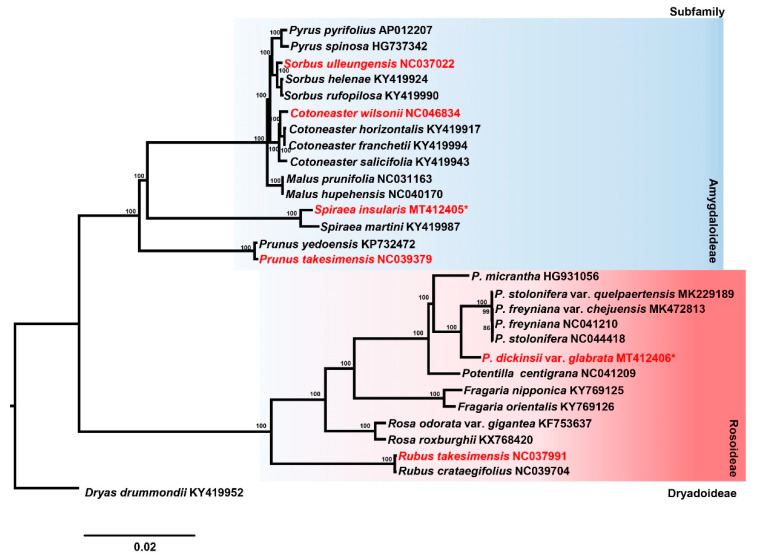
Maximum likelihood tree inferred from 29 representative taxa of Rosaceae. Bootstrap values based on 1000 replicates are shown on each node. Species indicated by red font are the six Rosaceae species endemic to Ulleung Island. * represent the newly assembled endemic plastomes of Rosaceae on Ulleung Island in this study.

Maximum likelihood analysis conducted on the best-fit model of “K3Pu + F + G4” enabled us to reveal phylogenetic positions among the endemic Rosaceae taxa on Ulleung Island (Figure 6). However, given that the phylogenetic tree was constructed based on only a partial representation of the entire Rosaceae family, the positions determined should be considered provisional and interpreted with caution. Nevertheless, our phylogenetic analysis of 29 representative plastomes within the rose family strongly supports the monophyly of Potentilla (100% bootstrap support) and the sister relationship between *Potentilla* and *Fragaria* (100% bootstrap support). We found that *P*. *dickinsii* var. *glabrata* was sister to a clade containing *P*. *freyniana*, *P*. *freyniana* var. *chejuensis*, *P*. *stolonifera*, and *P*. *stolonifera* var. *quelpaertensis*, whereas the clade of genus *Spiraea* (*S*. *insularis* and *S*. *martini*) is sister to a clade containing *Cotoneaster*, *Malus*, *Pyrus*, and *Sorbus* (Amygdaloideae; 100% bootstrap support). In addition, the monophyly of *Cotoneaster*, *Spiraea* and *Sorbus* was strongly supported, with a 100% bootstrap support value of each genus. *Cotoneaster wilsonii* showed sister relationships with two congeneric species, *C*. *horizontalis* and *C*. *franchetii*, while *Sorbus ulleungensis* showed sister relationships with congeneric *S*. *helenae* and *S*. *rufopilosa*. Lastly, we found that the genus *Prunus* represented the earliest diverged lineage within the subfamily Amygdaloideae and the monophyly of Amygdaloideae and Rosoideae was strongly supported with 100% bootstrap support.

## 3. Materials and Methods

### 3.1. Plant Sampling, DNA Isolation, and Plastome Sequencing/Annotation

To characterize plastome sequences among endemic species of the family Rosaceae on Ulleung Island, we collected samples from *Potentilla dickinsii* var. *glabrata* and *Spiraea insularis*, the two Ulleung endemic species in this family for which the plastomes have yet to be sequenced. Fresh leaves were collected from the Key-Chungsan Botanical Garden, which was specifically designated by the Ministry of Environment, Korea for the ex situ conservation of numerous native and endemic plant species from Ulleung Island. Voucher specimens were collected and deposited in the Ha Eun Herbarium of Sungkyunkwan University, Korea. Total DNA was isolated using a DNeasy Plant Mini Kit (Qiagen, Carlsbad, CA, USA) and sequenced using an Illumina HiSeq 4000 sequencer (Illumina, Inc., San Diego, CA, USA) at Macrogen Corporation (Seoul, Korea). A total of 33,832,832 and 29,396,622 paired-end reads (150 bp) were generated for *P. dickinsii* var. *glabrata* and *S. insularis*, respectively, and these were subsequently assembled de novo using Velvet v. 1.2.10 (EMBL-EBI, Cambridge, UK) with multiple k-mers [76]. tRNAs in the sequences were confirmed using tRNAscan-SE 2.0 (The Lowe lab, Santa Cruz, CA, USA) [77]. Annotation was conducted using Geneious R10 (Biomatters, Auckland, NewZealand) [78] and the annotated plastome sequences were deposited in the GenBank databank (with accession numbers of MT412406 and MT412405 for *P. dickinsii* var. *glabrata* and *S. insularis*, respectively). The annotated GenBank (NCBI, Bethesda, MD, USA) format sequence file was used to draw a circular map with the OGDRAW program v1.2 (CHLOROBOX, Postdam-Golm, Germany) [79].

### 3.2. Comparative Plastome Analysis

The complete plastomes of *P. dickinsii* var. *glabrata* and *S. insularis* were compared with those previously obtained for four Rosaceae species endemic to Ulleung, namely, *C. wilsonii* (NC046834), *Prunus takesimensis* (NC039379), *R. takesimensis* (NC037991), and *Sorbus ulleungensis* (NC03702). The six Rosaceae plastomes were aligned using MAFFT v. 7 [80] and adjusted manually using Geneious [78]. Using DnaSP v. 6.10 software [81], we performed a sliding window analysis, with a step size of 200 bp and window length of 800 bp, to determine plastome nucleotide diversity (*Pi*). The codon usage frequency was calculated using MEGA7 [82], yielding RSCU values [83], which are a simple measure of the non-uniform usage of synonymous codons in a coding sequence. For this purpose, we employed the DNA code used by bacteria, archaea, prokaryotic viruses, and in plant chloroplasts [84]. To predict putative RNA editing sites in the six plastomes, protein-coding genes were identified using the online program predictive RNA editor for plants (PREP) suite [85], with 22 genes used as references, based on a cut-off value of 0.8. Analyses based on the complete chloroplast genomes and the concatenated sequences of 75 common protein-coding genes among the studied species were conducted with MAFFT v. 7 [80], using Geneious R10 [78], and the Maximum likelihood phylogenetic tree was constructed with IQ-TREE ver. 1.4.2 [86]. To evaluate for natural selection pressure in the protein coding genes of the six plastomes, the site-specific model was performed using EasyCodeML [66] with CODEML algorithms [67]. Seven codon substitution models were investigated and compared to detect positively selected sites based on likelihood ratio tests (M0, M1a, M2A, M3, M7, M8, and M8a).

### 3.3. Phylogenetic Analysis

For the purposes of phylogenetic analysis, we analyzed the complete plastome sequences of 23 representative species from the family Rosaceae: seven species of *Potentilla*, including *P. centigrana* (NC041209), *P. freyniana* (NC041210), and *P. stolonifera* (NC044418); two species each from the genera *Fragaria*, *Malus*, *Prunus*, *Pyrus*, *Rosa*, and *Rubus*; one species each from the genera *Cotoneaster*, *Physocarpus*, and *Sorbus*. *Dryas drummondii* of the subfamily Dryadoideae was included as an outgroup species. The sequences of all species were aligned using MAFFT v. 7 [80] in Geneious [78]. Maximum likelihood analysis based on the best-fit model of K3Pu + F + G4 was conducted using IQ-TREE v. 1.4.2 [86], and non-parametric bootstrap analysis was performed with 1000 replicates.

## 4. Conclusions

In this study, we determined the complete plastome sequences of two species in the family Rosaceae (*Potentilla dickinsii* var. *glabrata* and *Spiraea insularis*) that are endemic to Ulleung Island, Korea. We found very little structural or organizational differences among the plastomes of six Rosaceae species endemic to this island. The frequency of codon usage was biased toward high RSCU values of U and A at the third codon position, and we found that the *ndh*B and *ndh*D genes are characterized by a high number of potential RNA editing sites. Comparative analysis among the six endemic Rosaceae species revealed three highly variable intergenic regions (*trnT*-UGU/*trnL*-UAA, *rpl32*/*trnL*-UAG, and *ndhF*/*rpl32*) and a single highly variable genic region (*ycf1*). These hotspot regions could be used to assess the genetic consequences of anagenetic speciation for endemic Rosaceae taxa on Ulleung Island. We also confirmed that a majority of the protein-coding genes (61 of 75) common to the chloroplast genomes of the endemic Rosaceae have been subjected to positive selection. Phylogenomic analysis based on selected Rosaceae plastomes supported the monophyly of the genus *Potentilla* and the sister relationship between *S. insularis* and a clade containing *Cotoneaster*, *Malus*, *Pyrus*, and *Sorbus*. The plastome resources reported in this study will enable us to gain a better understanding of the plastome evolution of insular endemics among the Rosaceae on Ulleung Island, as well as that of other members within the family Rosaceae.

## Figures and Tables

**Table 1:** Summary of the characteristics of the six endemic *Rosaceae* chloroplast genomes in Ulleung Island.

Taxa	*Potentilla dickinsii* var. *glabrata*	*Spiraea* *insularis*	*Cotoneaster* *wilsonii*	*Prunus* *takesimensis*	*Rubus* *takesimensis*	*Sorbus* *ulleungensis*
Total cpDNA size (bp)	155,524	158,637	159,999	157,948	155,760	159,632
GC content (%)	37.0%	36.9%	36.6%	36.7%	37.1%	36.5%
LSC size (bp)/GC content (%)	85,213/34.8%	86,997/34.8%	87,868/34.2%	85,959/34.6%	85,402/35.1%	88,003/34.2%
IR size (bp)/GC content (%)	25,827/42.8%	26,365/42.5%	26,399/42.6%	26,436/42.5%	25,781/42.8%	26,402/42.6%
SSC size (bp)/GC content (%)	18,657/30.7%	18,910/30.5%	19,335/30.3%	19,117/30.2%	18,750/31.1%	18,824/30.5%
Number of genes	131	132	131	131	131	131
Number of protein-coding genes	84	84	84	84	84	84
Number of tRNA genes	37	37	37	37	37	37
Number of rRNA genes	8	8	8	8	8	8
Number of duplicated genes	17	17	17	17	17	17
Number of single intron genes	15 (intron loss in *atpF*)	16	16	16	15 (intron loss in *atpF*)	16
Number of two intron genes	2	2	2	2	2	2
Accession Number	MT412406	MT412405	NC046834	NC039379	NC037991	NC03702

**Table 2:** Log-likelihood values of the site-specific models, with detected sites having dN/dS values > 1.

Gene Name	Models	np	ln L	Likelihood Ratio Test *p*-Value	Positively Selected Sites
*accD*	M8	14	−3164.733948	0.053131782	134 M 0.971 *
	M7	12	−3167.668928		
*ndhB*	M8	14	−2187.954632	0.000001793	371 S 0.974 *
	M7	12	−2201.186056		
*ndhD*	M8	14	−2896.605326	0.031007257	42 I 0.959 *
	M7	12	−2900.078860		
*ndhF*	M8	14	−4982.146816	0.000002084	643 S 0.952 *; 649 Q 0.990 *; 671 G 0.994 **
	M7	12	−4995.227947		
*rbcL*	M8	14	−2701.384653	0.000000000	86 H 0.980 *; 142 T 0.990 **; 247 C 0.999 **; 279 S 0.990 **; 475 L 0.998 **
	M7	12	−2729.389370		
*rps3*	M8	14	−1248.202111	0.000949733	97 T 0.952 *; 118 A 0.953 *
	M7	12	−1255.161441		

* *p* < 0.05; ** *p* < 0.01. np represents the degree of freedom.

## Supplementary Materials

Supplementary materials can be found at https://www.mdpi.com/1422-0067/21/14/4933/s1. Table S1. Predicted RNA editing sites in the complete chloroplast genomes of two Rosaceae species endemic to Ulleung Island.
